# The Preventive and Curative Treatment of Gas Gangrene by Mixed Serums

**Published:** 1919-01

**Authors:** 


**The Preventive and Curative Treatment of Gas Gangrene by Mixed Serums.** FRANCES Ivens, Surgeon-in-charge, Scottish Women's Hospital. *The British Medical Journal*, October 19, 1918.

The author speaks of the value of serum in the preventive and curative treatments of gas gangrene, and expresses her disappointment at seeing how little progress has been made in that line. She states that, in 1916, she used with success anti-perfringens serums prepared by Weinberg and Seguin at the Pasteur Institute as a curative measure in cases where the infection was established to the stage of intoxication and septicemia, and, from March 21st to September 6th, 1918, experimented the preventive use of anti-gangrenous serum on recently wounded arriving at hospital for a first operative treatment. A detailed account of the cases treated follows.

In the majority of cases the flora of gas gangrene is very complex. In addition to the Bacillus perfringens, other pathogenic organisms are found, such as the Vibrion septique and the Bacillus edematiens, preparing the ground for the streptococcus. Owing to such variety in the bacterial flora, mixed serums, or a polyvalent serum, active at once against the principal pathogenic microbes, were used :

(*a*) The mixed serums anti-perfringens, anti-Vibrion septique, anti-edematiens, prepared by Weinberg and Séguin.

(*b*) The polyvalent serum of Leclainche and Vallee.

(*c*) A combination of the two.

As was reported by P. Delbert on July 31st, 1918, in a series of 157 cases treated by the preventive use of mixed Weinberg serum, no death of gas gangrene occurred. During the period from March 21st, to September 6th, anti-gangrenous serums were given preventively in 433 cases, many of which already presented clinical signs of gas gangrene. These cases may be divided into three groups :

(1) 222 cases (126 fractures) : melange of 10 cc. each of anti-perfringens, anti-Vibrion septique, and anti-edematiens serum of M. Weinberg.

(2) 154 cases (110 fractures) : 30 cc. of Leclainche and Vallee polyvalent serum.

(3) 57 cases (34 fractures) : 30 cc. of melange (Weinberg serum) with 10 cc. of the Leclainche and Vallee polyvalent serum.

The serum was given subcutaneously in one pint of saline at the time of the operation.

*Group* 1. a) *Mortality.* Where the melange was given at or before the first operation, no case died from gas gangrene.

b) *Amputation.* Out of 14 cases in which the serum was administered at the time of amputation, 12 recovered. The remaining two died after a fortnight from streptococcal septicemia.

c) *Conservative treatment.* The administration of melange has, in a large number of cases, permitted conservative treatment instead of amputation.

10 cases have not been ascertained, owing to early evacuation of the patients.

*Group* 2. a) *Mortality.* 19 fatal cases, out of which 3 deaths were due to gas gangrene and 3 to gas gangrene with concurrent streptococcal septicemia.

b) *Amputation.* 15 amputations with 11 recoveries and 4 fatal results, out of which 2 were associated with streptococcal septicemia.

In the majority of cases, no severe streptococcal infection occurred during the period of preventive administration of the Leclainche and Vallee serum.

*Group* 3. Gangrene was present at the beginning of the treatment in 10 cases. Of these, massive gangrene developed only in one, 15 days after the preventive dose of serum had been given.

(a) *Mortality.* Two cases : one of massive gangrene, another from intestinal obstruction due to old appendicitis.

(b) *Amputation.* 3 cases : one for streptococcal infection, two for secondary hemorrhage.

Conservative treatment has been rendered possible in fractures of the dyaphysis and excellent results were attained in secondary operations through a preventive administration of Leclainche and Vallee serum.

The following conclusions are then drawn by the author :

(1) A powerful anti-gangrenous serum is of real value, as assistance to surgical treatment, in preventing the incidence of gas gangrene.

(2) If used in sufficient quantities, the serum is of great value as a disintoxicating agent in cases of advanced infection.

(3) The Leclainche and Vallee polyvalent serum has a marked influence on the after-history of cases with coincident streptococcal infections.

(4) Anaphylactic phenomena have been frequently averted through dilution of the serum by normal saline solution.

(5) When the isolation and identification of the special microbe is possible, the appropriate serum should be given, but owing to the length of time necessary for the carrying out of bacteriological examinations, immediate administration of the melange is recommended.

(6) Before secondary operations in infected cases, a further dose should be administered by preliminary fractional dose.

